# Predicting population coverage of T-cell epitope-based diagnostics and vaccines

**DOI:** 10.1186/1471-2105-7-153

**Published:** 2006-03-17

**Authors:** Huynh-Hoa Bui, John Sidney, Kenny Dinh, Scott Southwood, Mark J Newman, Alessandro Sette

**Affiliations:** 1 La Jolla Institute for Allergy and Immunology, Division of Vaccine Discovery, 3030 Bunker Hill Street, Suite 326, San Diego, CA 92109, USA; 2 IDM Inc., 5820 Nancy Ridge Drive, Suite 100, San Diego, CA 92121, USA

## Abstract

**Background:**

T cells recognize a complex between a specific major histocompatibility complex (MHC) molecule and a particular pathogen-derived epitope. A given epitope will elicit a response only in individuals that express an MHC molecule capable of binding that particular epitope. MHC molecules are extremely polymorphic and over a thousand different human MHC (HLA) alleles are known. A disproportionate amount of MHC polymorphism occurs in positions constituting the peptide-binding region, and as a result, MHC molecules exhibit a widely varying binding specificity. In the design of peptide-based vaccines and diagnostics, the issue of population coverage in relation to MHC polymorphism is further complicated by the fact that different HLA types are expressed at dramatically different frequencies in different ethnicities. Thus, without careful consideration, a vaccine or diagnostic with ethnically biased population coverage could result.

**Results:**

To address this issue, an algorithm was developed to calculate, on the basis of HLA genotypic frequencies, the fraction of individuals expected to respond to a given epitope set, diagnostic or vaccine. The population coverage estimates are based on MHC binding and/or T cell restriction data, although the tool can be utilized in a more general fashion. The algorithm was implemented as a web-application available at .

**Conclusion:**

We have developed a web-based tool to predict population coverage of T-cell epitope-based diagnostics and vaccines based on MHC binding and/or T cell restriction data. Accordingly, epitope-based vaccines or diagnostics can be designed to maximize population coverage, while minimizing complexity (that is, the number of different epitopes included in the diagnostic or vaccine), and also minimizing the variability of coverage obtained or projected in different ethnic groups.

## Background

T lymphocytes recognize a complex between a specific major histocompatibility complex (MHC) molecule and a particular pathogen-derived epitope. Thus, a given epitope will elicit a response only in individuals that express an MHC molecule capable of binding that particular epitope, explaining to a large extent the phenomenon known as "MHC restriction" [1]. In humans, MHC molecules are known as human leukocyte antigen (HLA) molecules and two different types exist: class I and class II. HLA class I molecules mostly bind peptides derived from the endogenous processing pathway, and their recognition is primarily associated with cytotoxic T lymphocytes (CTL), which are most important for antiviral and anticancer immunity responses. By contrast, HLA class II molecules bind peptides typically derived from the extracellular milieu, and they are important for helper T lymphocyte (HTL) responses, which regulate antibody and cytotoxic responses.

HLA molecules are extremely polymorphic. Over a thousand different HLA allelic variants have been defined to date [2]. Specific HLA alleles are expressed at dramatically different frequencies in different ethnicities [3,4]. Therefore, in the design and development of T-cell epitope-based diagnostics or vaccines, selecting multiple epitopes with different HLA binding specificities will afford increased coverage of the patient population. A pertinent goal, in this context, might be to identify optimal sets of HLA alleles with maximal coverages for different populations [5,6]. Extensive analyses by Longmate and coworkers [7] suggested that 90% population coverage of several ethnic groups can be achieved by targeting eleven different HLA molecules. However, 90% coverage of African and Asian ethnicities required four or more additional molecules. Dawson et al. also analyzed the problem [8] and concluded that to reach 80% coverage, 3 to 5 HLA molecules were required in a given ethnicity, but the actual HLA specificities required were different in different ethnic groups.

An important consideration in the process of epitope selection for a T-cell epitope-based diagnostic or vaccine is that the patient population coverage afforded by a given epitope set does not simply correspond to the sum of the coverage of the individual components. To calculate the coverage afforded by a given set of epitopes with multiple and/or overlapped HLA binding specificities, a more comprehensive approach, taking into account MHC binding and T cell recognition patterns, is required for this purpose. A suitable algorithm was previously utilized [9-11] but not described in detail. This method calculates the fraction of individuals predicted to respond to a given epitope or epitope set on the basis of HLA genotypic frequencies and on the basis of MHC binding and/or T cell restriction data. In this paper, we describe the algorithm and its implementation as a web application available to the public. We believe this is a useful tool to aid in the design and development of T-cell epitope-based diagnostics and vaccines intended to be effective across diverse populations.

## Implementation

For a given HLA gene locus, let {*m*_1_, *m*_2_, ..., *m*_*N*_} denote a set of MHC alleles, with each allele associated with a genotypic frequency *G*(*m*_*i*_) for a population or ethnic group. To account for 100% of alleles of a given locus, the total genotypic frequency (∑*G*(*m*_*i*_)) should add up to 1. If ∑*G*(*m*_*i*_) is less than 1, an unidentified HLA allele with a genotypic frequency equal to the residual (1 - ∑*G*(*m*_*i*_)) is added to the locus. If ∑*G*(*m*_*i*_) is greater than 1, the genotypic frequency of each *m*_*i *_allele of the locus is scaled down proportionately by dividing the frequency by ∑*G*(*m*_*i*_). Next, let {*e*_1_, *e*_2_, ..., *e*_*K*_} denote a set of epitopes with known MHC binding or restriction data. For each epitope *e*_*k*_, its restriction to an MHC allele *m*_*i*_, *e*_*k*_(*m*_*i*_), is defined as followed:

ek(mi)={0ifek is not restricted to mi1if ek is restricted to mi     (1).
 MathType@MTEF@5@5@+=feaafiart1ev1aaatCvAUfKttLearuWrP9MDH5MBPbIqV92AaeXatLxBI9gBaebbnrfifHhDYfgasaacH8akY=wiFfYdH8Gipec8Eeeu0xXdbba9frFj0=OqFfea0dXdd9vqai=hGuQ8kuc9pgc9s8qqaq=dirpe0xb9q8qiLsFr0=vr0=vr0dc8meaabaqaciaacaGaaeqabaqabeGadaaakeaacqWGLbqzdaWgaaWcbaGaem4AaSgabeaakiabcIcaOiabd2gaTnaaBaaaleaacqWGPbqAaeqaaOGaeiykaKIaeyypa0ZaaiqaaeaafaqaaeGacaaabaGaeGimaadabaWexLMBbXgBcf2CPn2qVrwzqf2zLnharyGvLjhzH5wyaGabaiaa=LgacaWFMbGaa8hiaiabdwgaLnaaBaaaleaacqWGRbWAaeqaaOGaeeiiaaIaeeyAaKMaee4CamNaeeiiaaIaeeOBa4Maee4Ba8MaeeiDaqNaeeiiaaIaeeOCaiNaeeyzauMaee4CamNaeeiDaqNaeeOCaiNaeeyAaKMaee4yamMaeeiDaqNaeeyzauMaeeizaqMaeeiiaaIaeeiDaqNaee4Ba8MaeeiiaaIaemyBa02aaSbaaSqaaiabdMgaPbqabaaakeaacqaIXaqmaeaacqqGPbqAcqqGMbGzcqqGGaaicqWGLbqzdaWgaaWcbaGaem4AaSgabeaakiabbccaGiabbMgaPjabbohaZjabbccaGiabbkhaYjabbwgaLjabbohaZjabbsha0jabbkhaYjabbMgaPjabbogaJjabbsha0jabbwgaLjabbsgaKjabbccaGiabbsha0jabb+gaVjabbccaGiabd2gaTnaaBaaaleaacqWGPbqAaeqaaaaaaOGaay5EaaGaaCzcaiaaxMaadaqadaqaaiabigdaXaGaayjkaiaawMcaaiabc6caUaaa@8AEA@

First, for each MHC allele (*m*_*i*_), a total number of epitope "hits", *H*(*m*_*i*_), was tabulated by adding the number of epitopes that are restricted to (or bound by) *m*_*i*_:

H(mi)=∑k=1Kek(mi)  (i=1,⋯,N)     (2).
 MathType@MTEF@5@5@+=feaafiart1ev1aaatCvAUfKttLearuWrP9MDH5MBPbIqV92AaeXatLxBI9gBaebbnrfifHhDYfgasaacH8akY=wiFfYdH8Gipec8Eeeu0xXdbba9frFj0=OqFfea0dXdd9vqai=hGuQ8kuc9pgc9s8qqaq=dirpe0xb9q8qiLsFr0=vr0=vr0dc8meaabaqaciaacaGaaeqabaqabeGadaaakeaacqWGibascqGGOaakcqWGTbqBdaWgaaWcbaGaemyAaKgabeaakiabcMcaPiabg2da9maaqahabaGaemyzau2aaSbaaSqaaiabdUgaRbqabaaabaGaem4AaSMaeyypa0JaeGymaedabaGaem4saSeaniabggHiLdGccqGGOaakcqWGTbqBdaWgaaWcbaGaemyAaKgabeaakiabcMcaPiabbccaGiabbccaGiabcIcaOiabdMgaPjabg2da9iabigdaXiabcYcaSiabl+UimjabcYcaSiabd6eaojabcMcaPiaaxMaacaWLjaWaaeWaaeaacqaIYaGmaiaawIcacaGLPaaacqGGUaGlaaa@51B1@

Next, for each possible diploid MHC combination (*m*_*i*_, *m*_*j*_), a phenotypic frequency *F*(*m*_*i*_, *m*_*j*_) was calculated based on individual allele genotypic frequency:

*F*(*m*_*i*_, *m*_*j*_) = *G*(*m*_*i*_) × *G*(*m*_*j*_)     (3)

For *n *MHC types, this corresponds to an *n *× *n *tabulation of the phenotypic frequency at which each specific pair of MHCs will be found in the population from which the MHC frequencies were derived. A similar table was also generated to contain the number of epitope hits per each of the MHC combinations *H*(*m*_*i*_, *m*_*j*_). In the case of heterozygous combinations, *H*(*m*_*i*_, *m*_*j*_) was calculated as the sum of the number of epitope hits associated with each of the two alleles, *H*(*m*_*i*_) + *H*(*m*_*j*_). This is because *m*_*i *_and *m*_*j *_are two different alleles, and therefore the number of epitope hits recognized by each allele in the combination is independent of each other. However, in the case of homozygous combinations which contain two identical alleles, the number of epitope hits was the same as the number of epitope hits of the given allele:

H(mi,mj)={H(mi)+H(mj)if i≠jH(mi)if i=j     (4).
 MathType@MTEF@5@5@+=feaafiart1ev1aaatCvAUfKttLearuWrP9MDH5MBPbIqV92AaeXatLxBI9gBaebbnrfifHhDYfgasaacH8akY=wiFfYdH8Gipec8Eeeu0xXdbba9frFj0=OqFfea0dXdd9vqai=hGuQ8kuc9pgc9s8qqaq=dirpe0xb9q8qiLsFr0=vr0=vr0dc8meaabaqaciaacaGaaeqabaqabeGadaaakeaacqWGibasdaqadaqaaiabd2gaTnaaBaaaleaacqWGPbqAaeqaaOGaeiilaWIaemyBa02aaSbaaSqaaiabdQgaQbqabaaakiaawIcacaGLPaaacqGH9aqpdaGabaqaauaabaqaciaaaeaacqWGibasdaqadaqaaiabd2gaTnaaBaaaleaacqWGPbqAaeqaaaGccaGLOaGaayzkaaGaey4kaSIaemisaG0aaeWaaeaacqWGTbqBdaWgaaWcbaGaemOAaOgabeaaaOGaayjkaiaawMcaaaqaaiabbMgaPjabbAgaMjabbccaGiabdMgaPjabgcMi5kabdQgaQbqaaiabdIeainaabmaabaGaemyBa02aaSbaaSqaaiabdMgaPbqabaaakiaawIcacaGLPaaaaeaacqqGPbqAcqqGMbGzcqqGGaaicqWGPbqAcqGH9aqpcqWGQbGAaaaacaGL7baacaWLjaGaaCzcamaabmaabaGaeGinaqdacaGLOaGaayzkaaGaeiOla4caaa@5DB7@

Based on the calculated *F*(*m*_*i*_, *m*_*j*_) and *H*(*m*_*i*_, *m*_*j*_) tables, a frequency distribution was assembled by tabulating the phenotypic frequencies of all MHC combinations associated with a certain number of epitope/HLA combination hits (*h*):

F(h)=∑i=1N∑j=1NF(mi,mj)I{H(mi,mj)=h}     (5),
 MathType@MTEF@5@5@+=feaafiart1ev1aaatCvAUfKttLearuWrP9MDH5MBPbIqV92AaeXatLxBI9gBaebbnrfifHhDYfgasaacH8akY=wiFfYdH8Gipec8Eeeu0xXdbba9frFj0=OqFfea0dXdd9vqai=hGuQ8kuc9pgc9s8qqaq=dirpe0xb9q8qiLsFr0=vr0=vr0dc8meaabaqaciaacaGaaeqabaqabeGadaaakeaacqWGgbGrcqGGOaakcqWGObaAcqGGPaqkcqGH9aqpdaaeWbqaamaaqahabaGaemOrayKaeiikaGIaemyBa02aaSbaaSqaaiabdMgaPbqabaGccqGGSaalcqWGTbqBdaWgaaWcbaGaemOAaOgabeaakiabcMcaPiabdMeajnaaBaaaleaacqGG7bWEcqWGibascqGGOaakcqWGTbqBdaWgaaadbaGaemyAaKgabeaaliabcYcaSiabd2gaTnaaBaaameaacqWGQbGAaeqaaSGaeiykaKIaeyypa0JaemiAaGMaeiyFa0habeaaaeaacqWGQbGAcqGH9aqpcqaIXaqmaeaacqWGobGta0GaeyyeIuoaaSqaaiabdMgaPjabg2da9iabigdaXaqaaiabd6eaobqdcqGHris5aOGaaCzcaiaaxMaadaqadaqaaiabiwda1aGaayjkaiaawMcaaiabcYcaSaaa@5DB8@

where I{H(mi,mj)=h}={1if H(mi,mj)=h0if H(mi,mj)≠h
 MathType@MTEF@5@5@+=feaafiart1ev1aaatCvAUfKttLearuWrP9MDH5MBPbIqV92AaeXatLxBI9gBaebbnrfifHhDYfgasaacH8akY=wiFfYdH8Gipec8Eeeu0xXdbba9frFj0=OqFfea0dXdd9vqai=hGuQ8kuc9pgc9s8qqaq=dirpe0xb9q8qiLsFr0=vr0=vr0dc8meaabaqaciaacaGaaeqabaqabeGadaaakeaacqWGjbqsdaWgaaWcbaGaei4EaSNaemisaGKaeiikaGIaemyBa02aaSbaaWqaaiabdMgaPbqabaWccqGGSaalcqWGTbqBdaWgaaadbaGaemOAaOgabeaaliabcMcaPiabg2da9iabdIgaOjabc2ha9bqabaGccqGH9aqpdaGabaqaauaabeqaciaaaeaacqaIXaqmaeaacqqGPbqAcqqGMbGzcqqGGaaicqWGibascqGGOaakcqWGTbqBdaWgaaWcbaGaemyAaKgabeaakiabcYcaSiabd2gaTnaaBaaaleaacqWGQbGAaeqaaOGaeiykaKIaeyypa0JaemiAaGgabaGaeGimaadabaGaeeyAaKMaeeOzayMaeeiiaaIaemisaGKaeiikaGIaemyBa02aaSbaaSqaaiabdMgaPbqabaGccqGGSaalcqWGTbqBdaWgaaWcbaGaemOAaOgabeaakiabcMcaPiabgcMi5kabdIgaObaaaiaawUhaaaaa@6092@ is an indicator function.

For calculation of coverage by epitope sets restricted to MHC alleles of multiple *k *different loci, a combined frequency distribution (*P*) as a function of epitope/HLA combination hits (*n*) was generated by merging *k *separate frequency distributions. This merging procedure is based on the assumption that linkages between MHC loci are in equilibrium, and was done as follows:

P(n)=∑h1≥1⋯∑hk≥1(∏i=1kFi(hi)I{∑i=1khi=n})     (6),
 MathType@MTEF@5@5@+=feaafiart1ev1aaatCvAUfKttLearuWrP9MDH5MBPbIqV92AaeXatLxBI9gBaebbnrfifHhDYfgasaacH8akY=wiFfYdH8Gipec8Eeeu0xXdbba9frFj0=OqFfea0dXdd9vqai=hGuQ8kuc9pgc9s8qqaq=dirpe0xb9q8qiLsFr0=vr0=vr0dc8meaabaqaciaacaGaaeqabaqabeGadaaakeaacqWGqbaucqGGOaakcqWGUbGBcqGGPaqkcqGH9aqpdaaeqbqaaiabl+UimnaaqafabaWaaeWaaeaadaqeWbqaaiabdAeagnaaBaaaleaacqWGPbqAaeqaaOGaeiikaGIaemiAaG2aaSbaaSqaaiabdMgaPbqabaGccqGGPaqkcqWGjbqsdaWgaaWcbaWaaiWaaeaadaaeWbqaaiabdIgaOnaaBaaameaacqWGPbqAaeqaaSGaeyypa0JaemOBa4gameaacqWGPbqAcqGH9aqpcqaIXaqmaeaacqWGRbWAa4GaeyyeIuoaaSGaay5Eaiaaw2haaaqabaaabaGaemyAaKMaeyypa0JaeGymaedabaGaem4AaSganiabg+GivdaakiaawIcacaGLPaaaaSqaaiabdIgaOnaaBaaameaacqWGRbWAaeqaaSGaeyyzImRaeGymaedabeqdcqGHris5aaWcbaGaemiAaG2aaSbaaWqaaiabigdaXaqabaWccqGHLjYScqaIXaqmaeqaniabggHiLdGccaWLjaGaaCzcamaabmaabaGaeGOnaydacaGLOaGaayzkaaGaeiilaWcaaa@672D@

where I{∑i=1khi=n}={1if ∑i=1khi=n0if ∑i=1khi≠n
 MathType@MTEF@5@5@+=feaafiart1ev1aaatCvAUfKttLearuWrP9MDH5MBPbIqV92AaeXatLxBI9gBaebbnrfifHhDYfgasaacH8akY=wiFfYdH8Gipec8Eeeu0xXdbba9frFj0=OqFfea0dXdd9vqai=hGuQ8kuc9pgc9s8qqaq=dirpe0xb9q8qiLsFr0=vr0=vr0dc8meaabaqaciaacaGaaeqabaqabeGadaaakeaacqWGjbqsdaWgaaWcbaWaaiWaaeaadaaeWbqaaiabdIgaOnaaBaaameaacqWGPbqAaeqaaSGaeyypa0JaemOBa4gameaacqWGPbqAcqGH9aqpcqaIXaqmaeaacqWGRbWAa4GaeyyeIuoaaSGaay5Eaiaaw2haaaqabaGccqGH9aqpdaGabaqaauaabeqaciaaaeaacqaIXaqmaeaacqqGPbqAcqqGMbGzcqqGGaaidaaeWbqaaiabdIgaOnaaBaaaleaacqWGPbqAaeqaaOGaeyypa0JaemOBa4galeaacqWGPbqAcqGH9aqpcqaIXaqmaeaacqWGRbWAa0GaeyyeIuoaaOqaaiabicdaWaqaaiabbMgaPjabbAgaMjabbccaGmaaqahabaGaemiAaG2aaSbaaSqaaiabdMgaPbqabaGccqGHGjsUcqWGUbGBaSqaaiabdMgaPjabg2da9iabigdaXaqaaiabdUgaRbqdcqGHris5aaaaaOGaay5Eaaaaaa@60DB@ is an indicator function, and *F*_*i*_(*h*_*i*_) is a phenotypic frequency associated with *h*_*i *_epitope/HLA combination hits of locus *i *calculated from equation 5.

The population coverage (*C*) or fraction of individuals projected to respond to the epitope set was then calculated as the sum of the combined phenotypic frequencies associated with at least one epitope hit/HLA combination:

C=∑n≥1P(n)     (7).
 MathType@MTEF@5@5@+=feaafiart1ev1aaatCvAUfKttLearuWrP9MDH5MBPbIqV92AaeXatLxBI9gBaebbnrfifHhDYfgasaacH8akY=wiFfYdH8Gipec8Eeeu0xXdbba9frFj0=OqFfea0dXdd9vqai=hGuQ8kuc9pgc9s8qqaq=dirpe0xb9q8qiLsFr0=vr0=vr0dc8meaabaqaciaacaGaaeqabaqabeGadaaakeaacqWGdbWqcqGH9aqpdaaeqbqaaiabdcfaqjabcIcaOiabd6gaUjabcMcaPaWcbaGaemOBa4MaeyyzImRaeGymaedabeqdcqGHris5aOGaaCzcaiaaxMaadaqadaqaaiabiEda3aGaayjkaiaawMcaaiabc6caUaaa@3DF6@

Based on equation 6, a histogram was generated to summarize the fraction of population coverage (*P*) as a function of the number of HLA/epitope combinations (*n*) recognized. A cumulative population coverage distribution frequency (*Y*) as a function of the number of HLA/epitope combinations (*n*) was also calculated:

Y(n)=∑x≥nP(x)     (8).
 MathType@MTEF@5@5@+=feaafiart1ev1aaatCvAUfKttLearuWrP9MDH5MBPbIqV92AaeXatLxBI9gBaebbnrfifHhDYfgasaacH8akY=wiFfYdH8Gipec8Eeeu0xXdbba9frFj0=OqFfea0dXdd9vqai=hGuQ8kuc9pgc9s8qqaq=dirpe0xb9q8qiLsFr0=vr0=vr0dc8meaabaqaciaacaGaaeqabaqabeGadaaakeaacqWGzbqwcqGGOaakcqWGUbGBcqGGPaqkcqGH9aqpdaaeqbqaaiabdcfaqjabcIcaOiabdIha4jabcMcaPaWcbaGaemiEaGNaeyyzImRaemOBa4gabeqdcqGHris5aOGaaCzcaiaaxMaadaqadaqaaiabiIda4aGaayjkaiaawMcaaiabc6caUaaa@41D8@

From this cumulative population coverage distribution of the whole epitope set, *PC*90, defined as the minimum number of epitope/HLA combination hits (*n*) recognized by 90% of the population, was determined as follow:

PC90=n+Y(n)−0.9Y(n)−Y(n+1)     (9),
 MathType@MTEF@5@5@+=feaafiart1ev1aaatCvAUfKttLearuWrP9MDH5MBPbIqV92AaeXatLxBI9gBaebbnrfifHhDYfgasaacH8akY=wiFfYdH8Gipec8Eeeu0xXdbba9frFj0=OqFfea0dXdd9vqai=hGuQ8kuc9pgc9s8qqaq=dirpe0xb9q8qiLsFr0=vr0=vr0dc8meaabaqaciaacaGaaeqabaqabeGadaaakeaacqWGqbaucqWGdbWqcqaI5aqocqaIWaamcqGH9aqpcqWGUbGBcqGHRaWkdaWcaaqaaiabdMfazjabcIcaOiabd6gaUjabcMcaPiabgkHiTiabicdaWiabc6caUiabiMda5aqaaiabdMfazjabcIcaOiabd6gaUjabcMcaPiabgkHiTiabdMfazjabcIcaOiabd6gaUjabgUcaRiabigdaXiabcMcaPaaacaWLjaGaaCzcamaabmaabaGaeGyoaKdacaGLOaGaayzkaaGaeiilaWcaaa@4C50@

where *Y*(*n*) ≥ 0.9 > *Y*(*n *+ 1). Because) *PC*90 was determined by data interpolation, it can be of any positive decimal value. Based on equation 9, if the population coverage is less than 90% or C=∑n≥1P(n)≡Y(1)<0.9
 MathType@MTEF@5@5@+=feaafiart1ev1aaatCvAUfKttLearuWrP9MDH5MBPbIqV92AaeXatLxBI9gBaebbnrfifHhDYfgasaacH8akY=wiFfYdH8Gipec8Eeeu0xXdbba9frFj0=OqFfea0dXdd9vqai=hGuQ8kuc9pgc9s8qqaq=dirpe0xb9q8qiLsFr0=vr0=vr0dc8meaabaqaciaacaGaaeqabaqabeGadaaakeaacqWGdbWqcqGH9aqpdaaeqbqaaiabdcfaqjabcIcaOiabd6gaUjabcMcaPaWcbaGaemOBa4MaeyyzImRaeGymaedabeqdcqGHris5aOGaeyyyIORaemywaKLaeiikaGIaeGymaeJaeiykaKIaeyipaWJaeGimaaJaeiOla4IaeGyoaKdaaa@42C5@, *PC*90 will be less than 1.

Additionally, the average number of epitope/HLA combination hits (A) recognized by the population is a weighted average and was calculated as follow:

A=∑n≥1n×P(n)     (10).
 MathType@MTEF@5@5@+=feaafiart1ev1aaatCvAUfKttLearuWrP9MDH5MBPbIqV92AaeXatLxBI9gBaebbnrfifHhDYfgasaacH8akY=wiFfYdH8Gipec8Eeeu0xXdbba9frFj0=OqFfea0dXdd9vqai=hGuQ8kuc9pgc9s8qqaq=dirpe0xb9q8qiLsFr0=vr0=vr0dc8meaabaqaciaacaGaaeqabaqabeGadaaakeaacqWGbbqqcqGH9aqpdaaeqbqaaiabd6gaUjabgEna0kabdcfaqjabcIcaOiabd6gaUjabcMcaPaWcbaGaemOBa4MaeyyzImRaeGymaedabeqdcqGHris5aOGaaCzcaiaaxMaadaqadaqaaiabigdaXiabicdaWaGaayjkaiaawMcaaiabc6caUaaa@4250@

## Results and discussions

The Population Coverage Calculation program was implemented as a Java servlet public web-application (see Availability and Requirements section). HLA allele (genotypic) frequencies were obtained from dbMHC database [12]. At present, dbMHC database provides allele frequencies for 78 populations grouped into 11 different geographical areas. In addition to the allele frequencies obtained from the dbMHC database, the Population Coverage Calculation program also accepts custom populations with allele frequencies defined by users. Multiple population coverages can be simultaneously calculated and an average population coverage is generated. Since MHC class I and MHC class II restricted T cell epitopes elicit immune responses from two different T cell populations (CTL and HTL, respectively), the program provides three calculation options to accommodate different coverage modes – (1) class I separate, (2) class II separate, and (3) class I and class II combined. For each population coverage, a histogram is generated to summarize the percentage distribution of individuals as a function of the number of epitope/HLA combinations recognized. A cumulative coverage distribution plot is also generated to determine the minimum number of epitope/HLA combinations recognized by 90% of the population (PC90). Finally, the average number of epitope/HLA combinations recognized by the population and coverages of individual epitope are also calculated.

It should be noted that when population coverages are projected from an epitope set restricted to alleles from multiple HLA loci, linkages between loci are taken into account. The overall population (phenotypic frequency), (*P*_*total*_), is mathematically derived as the sum of the individual locus' coverage corrected for the overlaps: S=∑k=1nn!k!(n−k)!
 MathType@MTEF@5@5@+=feaafiart1ev1aaatCvAUfKttLearuWrP9MDH5MBPbIqV92AaeXatLxBI9gBaebbnrfifHhDYfgasaacH8akY=wiFfYdH8Gipec8Eeeu0xXdbba9frFj0=OqFfea0dXdd9vqai=hGuQ8kuc9pgc9s8qqaq=dirpe0xb9q8qiLsFr0=vr0=vr0dc8meaabaqaciaacaGaaeqabaqabeGadaaakeaacqWGtbWucqGH9aqpdaaeWbqaamaalaaabaGaemOBa4MaeiyiaecabaGaem4AaSMaeiyiaeIaeiikaGIaemOBa4MaeyOeI0Iaem4AaSMaeiykaKIaeiyiaecaaaWcbaGaem4AaSMaeyypa0JaeGymaedabaGaemOBa4ganiabggHiLdaaaa@4072@, where *P*_*ij *_is the frequency of the *ij *haplotype, *P*_*ijk *_is the frequency of the *ijk *haplotype, etc... If gene linkage equilibrium is assumed, *P*_*ij *_can be calculated as the product of the individual allele phenotypic frequencies (*P*_*i *_× *P*_*j*_), and *P*_*ijk *_= *P*_*i *_× *P*_*j *_× *P*_*k*_, etc... This calculation is implicitly incorporated in our current algorithm (equation 6). However, if gene linkage is in disequilibrium, the frequency of a given haplotype is usually not equal to the product of their individual allele phenotypic frequencies, (*P*_*ij *_≠ *P*_*i *_× *P*_*j*_, *P*_*ijk *_≠ *P*_*i *_× *P*_*j *_× *P*_*k*_, ...). As a result, to account for linkage disequilibrium between HLA loci, complete data on haplotype frequencies must be known. Therefore, it would be difficult to factor in linkage disequilibrium at this time because linkage disequilibrium is known to be different in different ethnicities, and data regarding the specific disequilibrium in different ethnicities in general is not available or incomplete. As more comprehensive MHC linkage disequilibrium data becomes available, our method can be modified to incorporate this type of calculation.

Although the present program assumes linkage equilibrium between HLA loci, the impact of linkage disequilibrium, which is known to occur in the MHC region, on the calculated coverage is expected, in most contexts, to be minimal. For example, in the North American Caucasian population, the A1 and B8 antigens of HLA-A and -B loci, respectively, are known to be the strongest linked antigen pair with an observed haplotype frequency of 7.95% [13]. The genotypic frequencies of the A1 and B8 antigens are 15.18% and 9.41%, respectively [13]. Assuming the linkage between A1 and B8 antigens is in equilibrium, the overall population coverage calculated by the present program is 40.97%, and the individual population coverages by A1 and B8 antigens are 28.06% and 17.93%, respectively. The expected equilibrium frequency for the A1/B8 haplotype, in this case, is 5.03% (28.06% × 17.93%) which is 2.92% less than the observed frequency of 7.95%. Therefore, if linkage disequilibrium is considered, the overall population coverage will be 38.04% (28.06% + 17.93% - 7.95%). Thus, even for the most tightly linked A1/B8 haplotype in the Caucasian population, linkage disequilibrium, in this specific example, only accounted for less than 3% difference in the population coverage calculated by the present program. Furthermore, we have also investigated the deviations between the observed and expected equilibrium frequencies of 1012 HLA-A/-B haplotypes in the North American Caucasian population, based on available antigen- and haplotype-frequencies published by Mori *et al*. [14,15]. On average, the observed haplotype frequencies deviated from the expected equilibrium frequencies by approximately 0.58%. As a result, linkage disequilibrium is expected to impact the calculated population coverage, but the degree of the impact is expected to be negligible.

It should be pointed out that the calculations described herein can also be performed on data spreadsheets, but the process is laborious, error prone and also requires extensive immunological expertise. In our experience, a single calculation without the aid of this tool requires several hours to complete. To the best of our knowledge, at this time, there is no existing program that is publicly accessible as a web-resource that can offer the flexibility and range of utility similar to the Population Coverage Calculation program that we have developed. The present application represents a significant enhancement of the dbMHC database's utility by incorporating its compiled data of world-wide ethnic population frequencies to calculate HLA coverage for user-defined population subsets. The program is flexible by allowing the user to specify groups of related or unrelated ethnicities as well as specify the HLA alleles under consideration. Additional flexibility features include the implementation of separate calculations for both MHC Class I and Class II demarcated recognitions as they involve immune responses from two different populations of T cells – CTL and HTL, respectively. The output of the program was also specifically designed to be accessible to both specialists and neophytes in the field of MHC research. Therefore, having this tool publicly available is highly desirable. Additionally, in our future works, we plan to incorporate in the tool the ability to search for minimal epitope subset(s) within the given epitope set that will afford a specified population coverage level. This is not a trivial task due to a large number of possible epitope subsets (S) that has to be considered, S=∑k=1nn!k!(n−k)!
 MathType@MTEF@5@5@+=feaafiart1ev1aaatCvAUfKttLearuWrP9MDH5MBPbIqV92AaeXatLxBI9gBaebbnrfifHhDYfgasaacH8akY=wiFfYdH8Gipec8Eeeu0xXdbba9frFj0=OqFfea0dXdd9vqai=hGuQ8kuc9pgc9s8qqaq=dirpe0xb9q8qiLsFr0=vr0=vr0dc8meaabaqaciaacaGaaeqabaqabeGadaaakeaacqWGtbWucqGH9aqpdaaeWbqaamaalaaabaGaemOBa4MaeiyiaecabaGaem4AaSMaeiyiaeIaeiikaGIaemOBa4MaeyOeI0Iaem4AaSMaeiykaKIaeiyiaecaaaWcbaGaem4AaSMaeyypa0JaeGymaedabaGaemOBa4ganiabggHiLdaaaa@4072@ where *n *is the total number of epitopes and *k *is the number of epitopes in a subset. For example, for a set of 20 epitopes, there will be a total of 1,048,575 combinations of epitope subsets that needs to be evaluated. Therefore, a strategic searching approach must be devised to computationally accomplish this task. In summary, with the help of this Population Coverage Calculation program, epitope-based vaccines or diagnostics can be designed to maximize population coverage while minimizing complexity (that is, the number of different epitopes included in the diagnostic or vaccine), and also minimizing the variability of coverage obtained or projected in different ethnic groups.

## Conclusion

Herein, we have implemented a method to calculate projected population coverage of a T-cell epitope-based diagnostic or vaccine using MHC binding or T cell restriction data and HLA gene frequencies. The Population Coverage Calculation program was designed to be user friendly and flexible. Besides the compiled HLA gene frequencies currently provided, users can also supply their own tabulated HLA gene frequencies for calculation. Therefore, researchers can use this tool to perform coverage analyses on their specific patient populations. We plan to continuously update the compiled HLA gene frequencies as more data are available, and thus to provide researchers with a useful tool to aid in the design and development of effective T-cell epitope-based diagnostics and vaccines.

## Availability and requirements

Project name: Population Coverage Calculation

Project home page: 

Programming language: Java

Operating system: Fedora Linux

Other requirements: Apache Tomcat 5.5.12, MySQL 4.1

Web browser: Population Coverage Calculation program has been tested and shown to work with the following browsers: Firefox version 1.5 (PC and Mac OS X), Netscape version 8.0.4 (PC), Netscape version 7.2 (Mac OS X), Internet Explorer version 6.0 (PC), Internet Explorer version 5.2 for Mac (Mac OS X). Default security settings were used.

## Authors' contributions

HHB developed the computer algorithm and designed the web-resource. AS and JS contributed the calculation approaches. KD helped with programming and collecting HLA frequency data. SS and MN were involved in conceptualizing the calculation approaches. HHB wrote the manuscript, AS and JS edited the final version. All authors read and approved the manuscript.
